# Baseline Amino Acid Substitutions in the NS5A ISDR and PKR Binding Domain of Hepatitis C and Different Fibrosis Levels and Levels of Development of Hepatocellular Carcinoma in Patients Treated with DAAs

**DOI:** 10.3390/v12030255

**Published:** 2020-02-25

**Authors:** Stefania Paolucci, Antonio Piralla, Federica Novazzi, Alice Fratini, Renato Maserati, Roberto Gulminetti, Stefano Novati, Giorgio Barbarini, Paolo Sacchi, Annalisa De Silvestri, Fausto Baldanti

**Affiliations:** 1 Molecular Virology Unit, Microbiology and Virology Department, Fondazione IRCCS Policlinico San Matteo, 27100 Pavia, Italy; s.paolucci@smatteo.pv.it (S.P.); a.piralla@smatteo.pv.it (A.P.); novazzifederica@gmail.com (F.N.); alice.fratini01@universitadipavia.it (A.F.); 2 Institute of Infectious Diseases, University of Pavia, 27100 Pavia, Italy; r.maserati@smatteo.pv.it (R.M.); r.gulminetti@smatteo.pv.it (R.G.); s.novati@smatteo.pv.it (S.N.); 3 Division of Infectious and Tropical Diseases, Fondazione IRCCS Policlinico San Matteo, 27100 Pavia, Italy; g.barbarini@smatteo.pv.it (G.B.); p.sacchi@smatteo.pv.it (P.S.); 4 Clinical Epidemiology and Biometric Unit, Fondazione IRCCS Policlinico San Matteo, 27100 Pavia, Italy; A.DeSilvestri@smatteo.pv.it; 5 Department of Clinical, Surgical, Diagnostic and Pediatric Sciences, University of Pavia, 27100 Pavia, Italy

**Keywords:** fibrosis levels, hepatocellular carcinoma, HCV, DAA/direct-acting antivirals, ISDR and PKR-bd

## Abstract

Variations in the interferon sensitivity-determining region (ISDR) within the NS5A region were related to the development of hepatocellular carcinoma (HCC) in patients infected with hepatitis C virus (HCV). The aim of the study was to investigate a relationship between ISDR/PKR substitutions and their association with liver fibrosis or HCC development. A total of 316 patients infected with HCV and treated with DAAs were evaluated. HCV RNA was quantified and sequenced before treatment. The liver fibrosis stage was assessed by transient elastography and equalized to METAVIR scores. Multivariate analysis showed that ≥3 substitutions in ISDR and ≥6 in PKR-bd were significantly associated with advanced fibrosis. Advanced fibrosis was observed in patients with higher substitutions in ISDR and PKR-bd. A higher correlation between advanced fibrosis and a high frequency of ≥3 substitutions in ISDR and ≥6 in PKR-bd was observed in patients infected with genotype 2c. In addition, in a higher proportion of HCC patients, advanced fibrosis (40.4% vs. 88.2%; *p* < 0.001) and ≥6 substitutions in PKR-bd (15.4% vs. 41.2%; *p* = 0.01) was observed. In conclusion, a higher number of substitutions in ISDR and PKR-bd were associated with advanced liver fibrosis, suggesting a use of like predictors for progression in the liver damage. A significantly higher number of PKR-bd substitutions was observed in HCC patients; in particular, in patients infected with HCV genotype 2c.

## 1. Introduction

Hepatitis C virus (HCV) infection is one of the main causes of hepatocellular carcinoma (HCC). Therapy to eradicate HCV prevents the progression of liver fibrosis [1,2,3] and the development of HCC [4,5,6]. However, HCC can develop even in patients with chronic hepatitis C who achieve sustained virologic response (SVR) [7,8]. Previous studies have shown that liver fibrosis is strongly associated with the development of HCC after SVR [9,10]. Thus, patients at risk of HCC should continue to have close surveillance after SVR. In addition, it is important to determine which patients might have higher risk factors for HCC after SVR, and for how long they should be monitored.

Due to the recent introduction of direct-acting antiviral drugs (DAAs) for HCV, an increased number of patients with chronic hepatitis C have achieved SVR. However, the high degree of HCV genetic diversity may play an important role in the virus’s ability to evade the immune response and antiviral therapy’s selective pressure [11,12]. This variability could be focused on changes occurring at different positions in the NS3 protease, NS5B polymerase, and NS5A protein. Several studies have highlighted the influence of sequence heterogeneity within a particular region, especially in the NS5A, on the outcome of IFN-based therapy due to its relationship with IFN responsiveness [13]. NS5A modulates HCV replication through interactions with other viral proteins and host proteins to form the HCV replication complex and regulate host cell functions, including viral pathogenesis [14]. In particular, amino acid substitutions in the interferon sensitivity-determining region (ISDR) within the NS5A region have been related to advanced fibrosis and the development of HCC in patients infected with HCV [10,15]. In addition, it is known that ISDR is located within the protein kinase R-binding domain (PKR-bd) involved in a tumor suppressor function [16]. Other studies reported that NS5A resistance-associated substitutions (RASs) were associated with advanced liver fibrosis [13]. However, the relationship between NS5A substitutions and liver disease progression remains unclear. The aim of the present study is to investigate a potential relationship between the number of baseline ISDR/PKR-bd substitutions and the liver fibrosis stage and/or the development of HCC in SVR after treatment with DAAs.

## 2. Materials and Methods

### 2.1. Patient and Clinical Characteristics

This study was conducted in accordance with the principles of the Declaration of Helsinki and approved by the Institutional Review Board of the Fondazione IRCCS Policlinico San Matteo (protocol no. 20080009620, approval date: 11 March 2008). Written informed consent was obtained from all participants in the study. A total of 316 patients infected with HCV with genotypes 1a, 1b, 2c, 3a, and 4d referred to the Fondazione IRCCS Policlinico San Matteo, Pavia between January 2017 and April 2019 were evaluated in the study. All patients were treated with DAAs. In addition, 70/316 patients had previously experienced peg-IFN treatment. The liver fibrosis stage was assessed by transient elastography and equalized to METAVIR scores, and F0–F2 was classified as low fibrosis, while F3-F4 as the advanced fibrosis stage [10]. Patients were monitored for at least 3 months after the completion of treatment to evaluate SVR. The follow-up period was defined as the period from the confirmation of SVR to HCC development or the last visit to assess HCC. Currently, patients with advanced fibrosis before treatment are monitored after stopping therapy for the duration of their lives to see the possible progression in HCC.

### 2.2. HCV-RNA Quantification and Sequencing

Serum samples were collected at the baseline in all 316 patients treated with DAAs included in the study, and the HCV load was quantified by using the Abbott HCV-RNA assay (Abbott Park, IL, USA). HCV genotyping was performed using the Abbott RealTime HCV Genotype II assay. In addition, in order to resolve possible ambiguities during the genotyping, the NS3/NS5B region was sequenced to further subtype the HCV strains. Data were analyzed using the Blast program (http://blast.ncbi.nlm.nih.gov). Viral RNA was extracted from serum samples using the automatic Easy Mag extractor (Biomerieux, Lyon, France). HCV NS5A domain I (aa 1-406), NS3-protease (aa 1-181), and NS5B (aa 1-591) genes were amplified using a nested RT-PCR [17,18]. The amplified NS5A region included PKR-bd (from aa 237 to 302) and ISDR (from aa 237 to 302). Direct sequencing was performed by using an automatic sequencer (ABI PRISM 3130xl genetic analyzer DNA Sequencer, Applied Biosystems, Foster City, CA, USA) and the BigDye Terminator v1.1 Cycle Sequencing kit (Applied Biosystems) to evaluate the effect of the most represented viral quasispecies as already reported [10]. The RASs were defined according to the geno2pheno algorithm (http://hcv.geno2pheno.org/index.php) and other clinical and in vitro data for RAS interpretation [19,20,21]. Nucleotide sequences were aligned with MEGA 7.0 software and compared with the confirmed references: AF009606 for subtype 1a, D90208 for subtype 1b, JX227965 for subtype 2c, HM042073 for subtype 3a, and FJ462437 for subtype 4d. The reference strains have been chosen to be as homologous as possible to the HCV viral strains of the infected patients.

### 2.3. Statistical Analysis

Continuous variables (i.e., viral load) were compared using the Mann–Whitney *U* test for independent nonparametric data. Categorical variables were compared by the chi-square or Fisher’s exact test, as appropriate, and *p*-values of ≤0.05 were considered statistically significant. All these statistical analyses were performed using Graph Pad Prism software (version 5.00.288).

The cut-off values for the number of ISDR and PKR-bd amino acid substitutions were defined as three and six, respectively, by using ROC curve analysis. Univariate and multivariate analyses were performed under logistic regression models fitted for predicting high-grade fibrosis or HCC. These statistical analyses were performed using StataCorp USA 2017 (v15.1).

## 3. Results

Overall, only 9/316 (2.8%) patients failed to achieve SVR after treatment with DAAs. Development in HCC was observed in 17/316 (5.4%) patients within a year after treatment with DAAs. The evaluation of variants of the HCV NS5A-ISDR and PKR-bd genes was performed at pretreatment in all 316 patients. Overall, comparing ISDR and PKR-bd substitutions between SVR and failing-treatment patients, no significant differences were observed. In detail, the mean number of mutations observed in SVR and failing-treatment patients was comparable (1.85 ± 1.80 and 1.67 ± 1.32 in ISDR and 4.51 ± 2.42 and 4.67 ± 2.55 in PKR-bd, respectively *p* > 0.05). On the other hand, among all patients at baseline, a greater number of substitutions in both regions were observed in patients with advanced fibrosis levels. The mean number of mutations observed in the two regions of HCV patients with low and high fibrosis levels was comparable in ISDR (1.56 ± 1.28 and 2.20 ± 2.31; *p* = 0.13), while it was significantly different in PKR-bd (4.04 ± 1.74 and 5.07 ± 2.94; *p* = 0.006).

The results of univariate and multivariate analysis of factors associated with high fibrosis levels are shown in Table 1. In detail, 180/316 (56.9%) patients had low fibrosis values (F0–F2), and 136/316 (43.0%) patients had high fibrosis values (F3–F4). Univariate analysis showed that higher HCV RNA levels (*p* = 0.003), aspartate aminotransferase (AST) level (*p* < 0.001), γ-glutamyl transpeptidase (γ-GTP) levels (*p* < 0.001), male gender (*p* = 0.019), lower platelet (PLT) counts (*p* < 0.001), ≥3 substitutions in ISDR (*p* < 0.001), ≥6 substitutions in PKR-bd (*p* < 0.001), and PKR-bd with INS/DEL were associated with advanced fibrosis. According to multivariate analysis, higher HCV RNA level (*p* = 0.002), γGTP level (*p* = 0.002), lower platelet count (*p* < 0.001), ≥3 substitutions in ISDR (*p* = 0.007), and ≥6 in PKR-bd (*p* < 0.001) were identified as independent factors significantly associated with the development of advanced fibrosis. The number of substitutions observed in PKR-bd were significantly higher than those observed in ISDR (*p* < 0.001 vs. *p* = 0.007).

**Figure 1:**
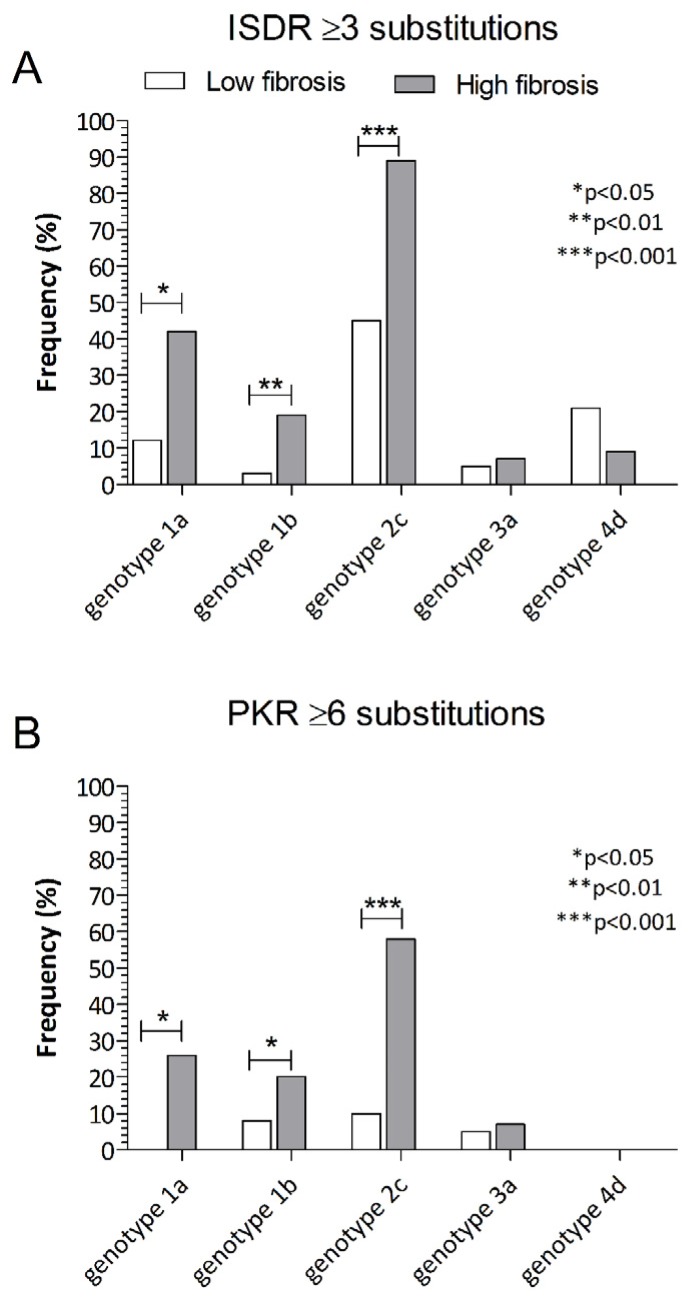
(**A**) Correlation between advanced fibrosis and ≥3 substitutions in interferon sensitivity-determining region (ISDR) according to genotype. (**B**) Correlation between advanced fibrosis and ≥6 substitutions in protein kinase R-binding domain (PKR-bd) according to genotype.

**Figure 2:**
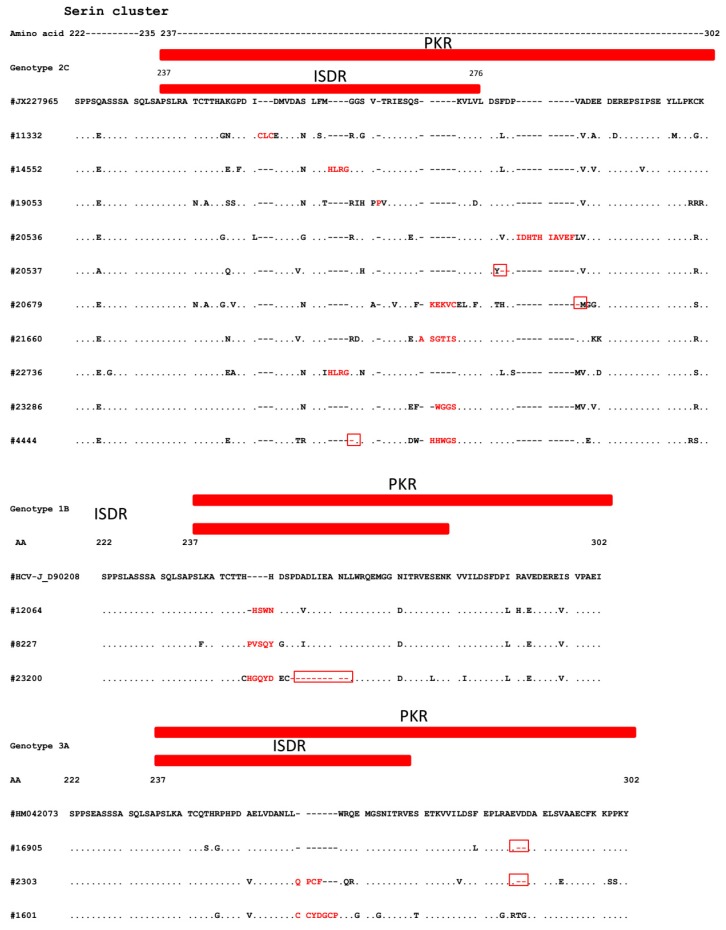
Sequence alignments of hepatitis C virus (HCV) with insertions/deletions in interferon sensitivity-determining region (ISDR) and direct-acting antiviral drugs (PKR-bd). Aminoacid insertions are in red (bold) and deletions are within red boxes.

Clinical characteristics analyzed according to substitutions in the ISDR and PKR-bd are shown in Table 2. Among patients with ≥3 substitutions in ISDR and ≥6 in PKR-bd, older age (*p* = 0.006, *p* < 0.001) higher AST levels (*p* = 0.03, *p* < 0.001), γGTP levels (*p* = 0.01, *p* = 0.003), ALT levels (*p* = 0.01) in PKR-bd, and advanced fibrosis (*p* < 0.001, *p* < 0.001) were more frequently observed. In particular, a higher proportion of patients with ≥3 substitutions in ISDR and ≥6 in PKR-bd was observed in patients infected with genotype 2c (*p* < 0.001 in both genes). In addition, according to genotypes, a higher correlation between advanced fibrosis and ≥3 substitutions in ISDR and ≥6 substitutions in PKR-bd was observed in patients infected with genotype 2c (*p* < 0.001, *p* < 0.001), followed by genotype 1b (*p* = 0.007, *p* = 0.04) and genotype 1a (*p* = 0.013, *p* = 0.002) (Figure 1). To note, HCV with insertions/deletions (INS/DEL) in PKR-bd was observed in 16/316 (5.0%) patients: 10 infected with genotype 2c, 3 with genotype 1b, and 3 with genotype 3a (Figure 2). Among them, a significantly greater proportion of patients had advanced fibrosis stage (11/16, 67.8%; *p* = 0.0037). In particular, significant lower HCV RNA levels were observed in patients carrying HCV with INS/DEL in ISDR (amino acid 237 to 270), compared to patients with HCV carrying INS/DEL in the remaining part of PKR-bd (amino acid 271 to 302) (*p* = 0.021).

The comparison of factors according to the development of HCC showed that older age (*p* = 0.05), lower HCV RNA levels (*p* < 0.001), higher alanine aminotransferase (ALT) level (*p* < 0.001), AST level (*p* < 0.001), γGTP level (*p* = 0.02), and lower platelet count (*p* < 0.001) were observed at the baseline of patients who developed HCC after treatment with DAAs (Table 3). In particular, in a higher proportion of patients who developed HCC advanced fibrosis (40.4% vs. 88.2%; *p* < 0.001) and ≥6 substitutions in PKR-bd (15.4% vs. 41.2%; *p* = 0.01) was observed. In addition, the proportion of patients who developed HCC was higher among patients infected with genotype 1b (47%) than with genotype 1a (0%), genotype 2c (29.4%), genotype 3a (23.5%), or genotype 4d (0%).

Nevertheless, according to genotype, a significantly higher proportion of virus-carrying ≥3 substitutions in ISDR and ≥6 in PKR-bd was observed in patients who developed HCC infected with genotype 2c (5/5, 100%) than with patients infected with genotype 1b (2/8, 25%) (*p* < 0.001). No virus carrying high substitution levels was observed among HCC patients infected with genotypes 1a, 3a, and 4d.

### Sequence Analysis of Other Viral Regions

Sequence analysis of the NS3 and NS5B regions was performed in all available samples to evaluate HCV variability in different genes. As expected, the proportion of amino acid changes detected in each region was variable, but no differences between samples from patients with low and high fibrosis levels were observed comparing NS3 and NS5B genes (data not shown).

## 4. Discussion

HCV eradication by DAA therapy reduces the progression of liver disease among patients with SVR. However, in a small proportion of them, a progression on HCC was observed [3,10,22]. The mechanisms and risk factors are still being investigated [23]. The optimal strategy for monitoring the incidence of advanced fibrosis and HCC remains unknown. Thus, it is important to determine the risk factors for liver damage progression after SVR. The association between substitutions in ISDR and development of HCC in patients who achieved SVR by IFN-based therapy has been reported in patients infected with HCV genotype 1b [10,15,24], which suggests that substitutions in ISDR might be a useful predictor for HCC together with higher γ-GTP level and older age. Here, the genetic variations in HCV NS5A-ISDR and PKR-bd of five different subtypes were evaluated. No differences were observed comparing SVR and treatment-failing patients. However, higher γ-GTP levels, lower PLT counts and plasma HCV RNA levels, as well as presence of higher level of substitutions in ISDR and in PKR-bd of HCV were associated with advanced liver fibrosis stage among all baseline treatment patients. In addition, a greater proportion of advanced fibrosis stage was observed among patients infected with HCV carrying INS/DEL in PKR-bd, as well as lower plasma HCV RNA levels being observed among patients infected with HCV carrying INS/DEL in ISDR compared to INS/DEL found in the rest of PKR-bd. As has already been hypothesized for the duplication of the V3 domain in NS5A, a higher number of substitutions in PKR-bd might alter protein functionality [25]. In fact, it is known that PKR can affect the pathogenesis of malignancies leading to cell growth [26,27] and may induce HCC cell proliferation with HCV infection [28]. Since the NS5A region suppresses PKR function, amino acid changes in ISDR and PKR-bd affect the binding and repression of PKR [16], thus determining a progression in the liver damage and hepatocarcinogenesis. NS5A of HCV genotype 1b carrying an ISDR with amino acid substitutions was associated with a reduced efficiency of infectious virus production in an in vitro human hepatocyte model determining a lower propagation ability [29,30]. Since lower plasma HCV RNA levels were observed among patients infected with HCV carrying higher substitutions in PKR-bd and carrying INS/DEL in ISDR, our hypothesis is that the mutated PKR-bd virus trapped in hepatocytes might indirectly determine a role in hepatocyte damage that could trigger a process of degeneration in HCC. Thus, higher levels of substitutions in the ISDR and even more in PKR-bd might be a useful predictor for advanced liver fibrosis and possibly for progression in HCC.

Among all patients infected with HCV carrying ≥3 substitutions in ISDR and ≥6 in PKR-bd, advanced fibrosis was observed in a higher proportion of patients infected with genotype 2c followed by patients infected with genotype 1b and genotype 1a. Overall, a higher proportion of patients who developed HCC had advanced fibrosis and ≥6 substitutions in PKR-bd compared to patients who did not develop HCC. Among pegIFN/RBV-treated patients, the highest percentage of subjects developing HCC was infected with HCV genotype 1b [31], and still now, among patients treated with DAAs, the higher proportion is infected with genotype 1b. However, among patients who developed HCC, a significant higher proportion infected with HCV genotype 2c had (i) higher substitutions in ISDR and PKR-bd, compared to patients infected with other genotypes, (ii) greater variability associated with advanced fibrosis, and (iii) a higher number of patients who developed HCC carried high numbers of PKR-bd substitutions at the baseline samples. Thus, it would be possible that over time this will lead to a change on the incidence of a greater frequency of development in HCC among patients infected with this subtype. However, further investigations are needed to assess (i) how many other recently treated patients will still develop HCC in a longer follow-up, (ii) the correlation between ISDR or PKR-bd substitutions and development in HCC, (iii) the correlation between advanced fibrosis after SVR and HCC development, and (iv) which genotype will be the most prone to develop HCC. With this perspective, it would be necessary to consider in the patient’s follow-up greater controls in assessing the state of the liver for longer periods and in particular considering, before starting DAA treatment, the presence of a greater number of ISDR/PKR-bd substitutions as a prognostic marker for advanced fibrosis and HCC.

## Figures and Tables

**Table 1:** Univariate and multivariate analysis of factors associated with high fibrosis levels.

	Univariate				Multivariate	
	F0–F2 (180)	F3–F4 (136)	OR (95%IC)	*p* Value	OR (95%IC)	*P* Value
Age in years (SD)	62.24 (±13.0)	63.69 (±14.0)	1.0 (0.9–1.1)	0.34		
Gender (male) (%)	86 (47.7)	83 (61.0)	1.7 (1.1–2.7)	**0.019**		
ALT (IU/L) (IQR)	30 (20–46.5)	62.5 (39.5–100)	1.0 (0.9–1.0)	0.16		
AST (IU/L) (IQR)	28 (23–40)	58.5 (34.5–96.5)	1.0 (1.0–1.0)	**<0.001**		
PLT (10^4^/mm^3^) (SD)	199.78 (±72.9)	160.25 (±59.6)	0.9 (0.9–0.9)	**<0.001**	0.99 (0.98–0.99)	**<0.001**
γGTP (IU/L) (IQR)	28.5 (18–46)	65 (34–105)	1.0 (1.0–1.0)	**<0.001**	1.01 (1.00–1,01)	**0.002**
Log_10_ HCV load (UI/mL) (SD)	5.71 (±0.89)	5.4 (±0.95)	0.7 (0.5–0.8)	**0.003**	0.65 (0.49–0.85)	**0.002**
Genotype 1a (%)	33 (18.3)	19 (13.9)	0.6 (0.3–1.1)	0.45		
Genotype 1b (%)	57 (31.6)	45 (33.1)	0.7 (0.3–1.4)			
Genotype 2c (%)	57 (31.6)	38 (27.9)	0.8 (0.4–1.4)			
Genotype 3a (%)	19 (10.5)	23 (16.9)	1.5 (0.7–3.1)			
Genotype 4d (%)	14 (7.7)	11 (8.1)	0.9 (0.4–2.4)			
ISDR ≥ 3 (%)	36 (20)	54 (39.7)	2.6 (1.5–4.3)	**<0.001**	2.14 (1.2–3.7)	**0.007**
PKR-bd ≥ 6 (%)	12 (6.6)	38 (27.9)	5.4 (2.7–10.8)	**<0.001**	4.8 (2.2–10.2)	**<0.001**
Ins/del (%)	5 (2.7)	11 (8.0)	3.1 (1.0–9.1)	**0.04**		

ALT, alanine aminotransferase; AST, aspartate aminotransferase; PLT, platelet; γGTP, γ-glutamyl transpeptidase; SD, standard deviation in mean analysis; IQR, interquartile range in median analysis. In bold are the significant values.

**Table 2:** Clinical characteristics according to the number of substitutions in the ISDR and PKR-bd.

	ISDR Mutations			PKR-bd Mutations		
	<3 (226)	≥3 (90)	*p* Value	<6 (235)	≥6 (81)	*p* Value
Age in years (SD)	61.5 (±13)	66 (±14)	**0.006**	60.6 (±13)	69.3 (±13)	**<0.001**
Gender (male) (%)	119 (52.7)	52 (56.5)	0.70	128 (54.5)	41 (50.6)	0.61
ALT (IU/L) (IQR)	39.5 (25–64)	44.5 (23–87)	0.31	38 (23–64)	53 (25–98)	**0.01**
AST (IU/L) (IQR)	33 (26–55)	48.5 (25.3–78.8)	**0.03**	32 (25–52)	52 (29–91)	**<0.001**
PLT (10^4^/mm^3^) (SD)	187.4 (±73.1)	171.2 (±61)	0.13	185.3 (±71.1)	175.3 (±67.2)	0.50
γGTP (IU/L) (IQR)	36.5 (22–63)	57.5 (24.3–108.3)	**0.01**	36 (21–64)	59 (25–100)	**0.003**
Log_10_ HCV load (UI/mL) (SD)	5.6 (±0.9)	5.5 (±0.9)	0.71	5.7 (±0.9)	5.3 (±0.9)	**0.002**
Genotype 1a (%)	40 (17.7)	12 (13.3)	**<0.001**	45 (19.1)	7 (8.6)	**<0.001**
Genotype 1b (%)	91 (40.3)	11 (12.2)		74 (31.5)	28 (34.6)	
Genotype 2c (%)	35 (15.5)	60 (66.7)		53 (22.5)	42 (51.8)	
Genotype 3a (%)	39 (17.2)	3 (3.3)		39 (16.6)	3 (3.7)	
Genotype 4d (%)	21 (9.3)	4 (4.4)		24 (10.2)	1 (1.2)	
Advanced fibrosis (F3-F4) (%)	82 (36.3)	54 (60.1)	**<0.001**	82 (34.9)	54 (66.7)	**<0.001**

ALT, alanine aminotransferase; AST, aspartate aminotransferase; PLT, platelet; γGTP, γ-glutamyl transpeptidase; SD, standard deviation in mean analysis; IQR, Interquartile range in median analysis. In bold are the significant values.

**Table 3:** Baseline clinical characteristics of patients treated with direct-acting antiviral drugs (DAAs) according to development in hepatocellular carcinoma (HCC).

	NonHCC	HCC	*p* Value
Number of patients	299	17	
Age in years (SD)	62.5 (±13.2)	66 (±10)	**0.05**
Gender (male) (%)	158 (52.84)	11 (64.7)	0.07
ALT (IU/L) (IQR)	38 (23–67)	59 (52–102)	**<0.001**
AST (IU/L) (IQR)	34.5 (25–62)	70 (48–105)	**<0.001**
PLT (10^4^/mm^3^) (SD)	185 (±70)	136 (±56)	**<0.001**
γGTP (IU/L) (IQR)	38 (21–71)	76 (37–114)	**0.02**
Log_10_ HCV load (UI/mL) (SD)	5.62 (±0.91)	4.76 (±0.9)	**<0.001**
Genotype 1a (%)	52 (17.4)	0 (0)	**<0.001**
Genotype 1b (%)	94 (31.4)	8 (47.0)	
Genotype 2c (%)	90 (30.1)	5 (29.4)	
Genotype 3a (%)	38 (12.7)	4 (23.5)	
Genotype 4d (%)	25 (8.3)	0 (0)	
ISDR ≥3 (%)	83 (27.7)	7 (41.2)	0.27
PKR-bd ≥6 (%)	46 (15.4)	7 (41.2)	**0.01**
Ins/del (%)	16 (4.4)	0 (0)	0.37
Advanced fibrosis (F3–F4) (%)	121 (40.4)	15 (88.2)	**<0.001**

ALT, alanine aminotransferase; AST, aspartate aminotransferase; PLT, platelet; γGTP, γ-glutamyl transpeptidase; SD, standard deviation in mean analysis; IQR, interquartile range in median analysis. In bold are the significant values.
